# The League of Mercy

**Published:** 1909-01-09

**Authors:** 


### THE LEAGUE OF MERCY.

We regret to state that in the list of the sums received from the districts of the League of Mercy in 1908, published on page 335 of our issue of December 26 last, the following districts were accidentally omitted :—

Hitchin £104, as against £48 last year, an increase of £56.
Enfield £96, as against £88 last year, an increase of £8.
Gravesend £88, as against £99 last year, but further sums are promised.

Each of the above districts has done excellent work during the year in spite of many difficulties, and we much regret the oversight which caused their returns to be omitted in the list we previously published.

The return for Hampstead was £313, as against £343, and for Mid-Essex £276, as against £237.

